# Morphological, molecular characterization, plant pathogenicity and biocontrol of *Cladosporium* complex groups associated with faba beans

**DOI:** 10.1038/s41598-021-93123-w

**Published:** 2021-07-09

**Authors:** Eman Gamal Abd Elnaser Mohamed El-Dawy, Youssuf Ahmed Gherbawy, Mohamed Ahmed Hussein

**Affiliations:** 1 Botany and Microbiology Department, Faculty of Science, South Valley University, Qena, Egypt; 2 Applied and Environmental Microbiology Center, South Valley University, Qena, Egypt

**Keywords:** Microbiology, Molecular biology

## Abstract

*Vicia faba* (faba bean) is one of the most significant leguminous crops. The faba bean is specialized by maximum nutritional value, in energy and protein content, which leads it to be suitable for food and feed production. Diseases caused with fungi are amongst the biotic factors responsible for decreasing in faba bean yields. In this work, *Cladosporium* isolates were recorded in cultivated faba bean leaves and pods collected from markets in Qena, Upper Egypt; morphological features and molecular characterization based on actin gene were performed. The ability of the pathogens to cause disease in faba bean seedlings and the biocontrol method to avoid the pathogenic effect of *Cladosporium* were determined. Results showed that *Cladosporium* is the main genera isolated from faba beans, and the morphological criteria showed presence of three species complex groups of *Cladosporium* (*C. cladosporioides*, *C. herbarum* and *C. sphaerospermum*) and the confirmation with molecular characterization revealed the existence of four species in the three groups. All the 26 tested strains of *Cladosporium* were able to cause leaf lesions on *Vicia faba* seedlings with different levels. *Chaetomium globosum* is a biocontrol agent could inhibit the growth of the majority strains of *Cladosporium*.

## Introduction

*Vicia faba* L. (faba bean) is a main legume species; growing in winter season in the area of North Nile Delta^1^. It has high protein content (24–30%), nutritional value, and high energy, and the faba bean is grown for animal feed and human food^2,3^. The quality and yield of faba bean seeds are affected by fungal diseases, which are among the biotic factors cause major yield losses in faba beans, by decreasing the growth of plants, in excessive cases leading to wilt and plant death^4^. Some fungal pathogens can cause the closure of seed plantations by epidemic outbreaks^5^.

*Cladosporium* represents one of the considerable genera of Ascomycota, Pezizomycotina, Dothideomycetes, Pleosporomycetidae, Capnodiales, Cladosporiaceae^6^. It comprises species that are among the most prevalent molds isolated from different environments in the world^7^. Many species are plant pathogens, and others are found as spoilage factors or contaminants in food or industrial products, as well as being frequently associated with asthmatic sickness and endophytic fungi^7,8^.

The genus of *Cladosporium* comprised three major species complexes, mainly based on morphology: *C. cladosporioides*, *C. herbarum* and *C. sphaerospermum* species complexes and used for practical purposes. Morphological features describing the three species complexes have been reported by Bensch et al.^9–11^. Based on the morphology, majority of the *Cladosporium* species can be referred to one of the three species complexes. Otherwise, many studies led to the introduction of subgenera due to the discovery of three diverse complexes: *C. cladosporioides*^12^, *C. herbarum*^13^ and *C. sphaerospermum*^14,15^, each of complex group comprised species that are differentiated morphologically and genetically. Species appearing clear morphological differences may be belonging to different species complexes and even genetically they are usually discriminated and form separate clades^9,16^. In order to identify and distinguish closely related species that showing similar morphological features, molecular sequences are used to accurate identification. However, ITS sequencing is often not sufficient to differentiate closely related species and morphologically similar within complexes. A few researchers choose to use a multilocus DNA sequences dependent on actin, ITS, calmodulin, translation elongation factor 1-a and histone H3 because they show a high discrimination of closely related species^12,13^.

The aim of this study was to identify of *Cladosporium* species by morphological characterization and confirmed by molecular sequences, determine the pathogenic effect of *Cladosporium* isolates on faba beans and apply biocontrol method in order to use preventive measures, so avoid biological damage and economical losses (Supplementary Information).

## Materials and methods

### Isolation, and determination of morphological criteria

Leaves and pods of cultivated *Vicia faba* infected with dark fungi were collected from farms and markets in Qena, Egypt during winter. The dilution- and baiting-plate methods were used for the estimation of mycobiota associated with leaves and pods of *Vicia faba* as described by Christensen^17^ and employed by Abdel-Hafez et al.^18,19^. In dilution- plate method, one gram of leaf or pod sample was removed, transferred to 100 ml sterile distilled water and shacked by hands for 10 min. One ml aliquot was inoculated into sterile petri dish, followed by adding 15 ml liquefied DG18 medium (g/l: 10 glucose; 5 peptone; 1 KH_2_PO_4_; 0.5 MgSO_4_; 0.002 dichloran; 220 glycerol; 0.025 rosebegal; 0.1 chloramphenicol)^20^. The plates were carefully rotated clockwise and anticlockwise to ensure distribution of homogenates, and then incubated at 28 °C for 7 days. In baiting-plate method: firstly, the samples were rinsed gently in running water to remove dust and debris. Then, samples were cut into 10 mm in diameter and dried between sterile filter paper. Finally, four segments were inoculated on DG18 plate. The plates incubated at 28 °C for 7 days then the developing fungi were counted and identified morphologically based on macro- and microscopic characters on PDA medium according to Bensch et al.^9^.

### DNA extraction from *Cladosporium* isolates

The isolation of DNA from *Cladosporium* mycelium was carried out according to a CTAB protocol^21^. Isolates were grown on PDA plates for 2–3 days at 28 °C. The mycelium was collected by using a spatula, milled with 700 µl 2 x CTAB buffer in a mortar, transferred to Eppendorf tubes and vortex for 2 min; incubation at 65 °C for 60–80 min was done, and then 700 µl of chloroform was mixed briefly. The mixture was centrifuged at 15,000 rpm for 10 min, the supernatant was transferred to a new tube, mixed briefly by 600 µl isopropanol, and chilled to 20 °C, followed by another centrifugation at 15,000 rpm for 5 min. The supernatant was removed and the settling pellet was washed by 1000 µl of 70% ethanol; another centrifugation for 3 min at 15,000 was carried out, drying the pellet and dissolved it in 50 µl TE (pH 7.5: 10 mM Tris, 1 mM EDTA) buffer. The DNA quality was tested by electrophoresis on a 1.4% agarose gel, which was supplied by ethidium bromide, and visualized by UV trans-illumination.

### PCR amplification and sequence analysis

For identifying *Cladosporium* isolates at species level, PCR amplification of partial gene sequences of the actin gene (ACT) were performed. PCR amplification for ACT gene was carried out by primers ACT-512F: ATGTGCAAGGCCGGTTTCGC and ACT-783R: TACGAGTCCTTCTGGCCCAT^9,22^. PCR was done in PCR tubes containing a total volume of 25 μl: 5 μl of the master mix (buffer, dNTP, Taq DNA polymerase, 2 mM MgCl2), 1 μl of the template DNA, 0.5 μl of both forward and reverse primers and 18 μl of PCR water. Amplification of actin gene was performed in a thermal cycler (Flexigene, Techne, Cambridge, UK). Amplification condition was: initial denaturation step at 95 °C for 8 min followed by 35 cycles of denaturation at 95 °C for 30 s, annealing at 48 °C for 30 s and extension at 72 °C for 1 min, with a final extension step at 72 °C for 5 min. Water was used as a negative control.

### Phylogenetic analysis

The sequences of actin gene were edited by Chromas Lite program. The sequence results were compared with other fungal sequences in NCBI’s GenBank sequence database to identify species by using a blast search. An alignment of the sequences was carried out with the CLUSTAL W program^23^. Sequences were submitted to GenBank on the NCBI website (http://www.ncbi.nlm.nih.gov). The resulting sequences were deposited in GenBank with accession numbers listed in Table 2. Phylogenetic reconstruction of the partial actin sequences was carried out using MEGA6 software package^24^. Maximum likelihood analyses were conducted using the Tamura–Nei model^25^. To detect the support for each clade, a bootstrap analysis was performed with 1000 replications. *Alternaria alternata* MK 896856.1 was used as outgroup.

### Pathogenicity effect of *Cladosporium* strains

Twenty-six strains of four species of *Cladosporium*, collected from diseased leaves and pods of *Vicia faba*, were tested for their pathogenicity effect on *Vicia faba* seedlings as method described with Berner et al.^26^. *Vicia faba* seeds were grown in a glasshouse. Firstly, sandy clay soil was sterilized by using autoclave 1:2 (w/w). Then three kg of sterilized soil was placed in pots with 200 mm in diameter. By using 0.1% mercuric chloride, seed surface was done for 2 min and washed several times with sterile water. Each pot was sown by three seeds and daily irrigated daily by water until seedlings’ appearance. Spore suspensions of *Cladosporium* species strains were used for inoculation of plants. The isolates were cultured on potato dextrose agar medium. The pathogenicity of tested species was assayed after 20 days of seeds grown by spraying of leaves. Three replicates were carried out for each isolate and control plant was sprayed by sterile distilled water. Spots on the leaf surface of plants were recorded after 6–10 days of inoculation.

### Inhibition the growth of *Cladosporium *strains by *Chaetomium globosum*

*Chaetomium globosum* (isolated as an endophytic fungus from *Ziziphus lotus* leaves) was tested for antagonism against *Cladosporium* strains using the dual culture technique. The culture medium was potato dextrose agar, PDA (g/l: 200 potato; 20 glucose; 15 agar)^27^. The medium was poured into petri dishes. A single disc with 8 mm of *Cladosporium* strain and *C. globosum* colonies were plated in the same petri dish. The plates were incubated at 28 °C for 7 days. Triplicates were used for each *Cladosporium* isolate. Radial growth reduction was calculated according to the formula of mycelial inhibition (%) = [(*r *‒ *r*^*−*^)/*r*] × 100, where *r* (mm) is the growth of *Cladosporium* from the center of the colony disc towards the edge of the petri dish and *r*^*−*^ (mm) is the growth of the *Cladosporium* from the center of the colony towards the center of the tested *C. globosum*^27^.

### Statistics

Statistical analyses were carried out on repeated measurements by one-way analysis of variance (ANOVA) by using Prism Version 6.0 (GraphPad Software, Inc., San Diego, CA, USA), followed by Bonferroni’s multiple comparison test^28^.

### Research involving human participants and/or animals

We declare that no human or animal were involved during the research.

### Permission statement

This is a permission from South Valley University to collect samples of cultivated faba beans from fields of the South Valley University and markets in Qena, were complied with relevant institutional, national, and international guidelines and legislation and transferring them safely to mycological analysis to South Valley University, Faculty of Science, Botany and Microbiology department, Applied and Environmental Microbiology Center.

## Results

### Mycobiota associated with *Vicia faba* leaves and pods

**Figure 1:**
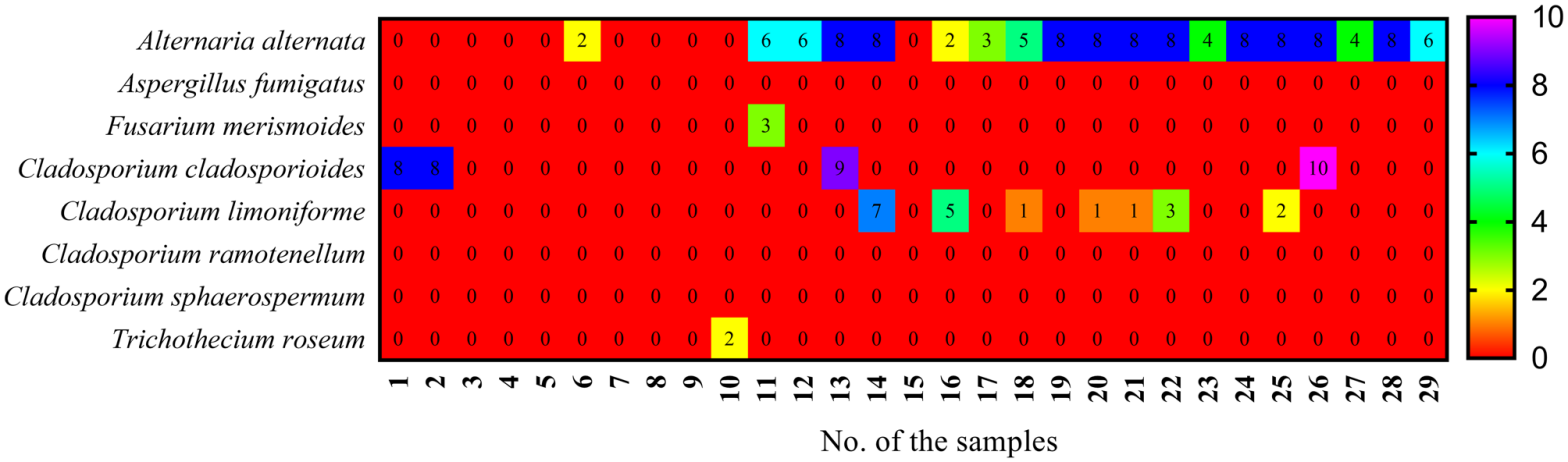
Fungal counts of species isolated from infected *Vicia faba* leaves and pods with baiting-plate method on DG18 medium at 28 °C for 7 days.

**Figure 2:**
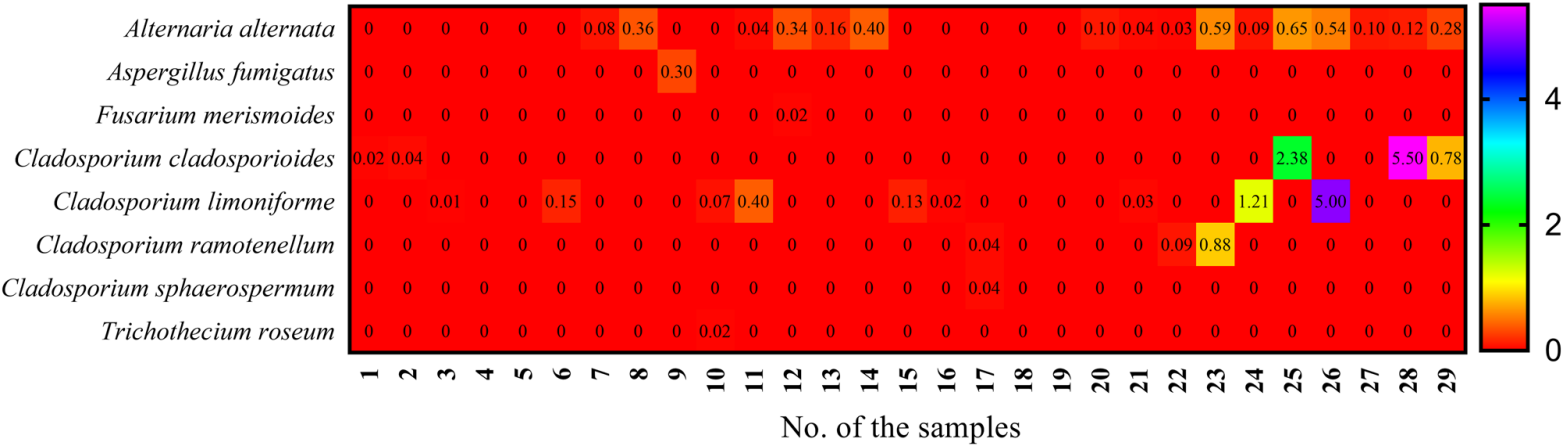
CFU of species isolated from infected *Vicia faba* leaves and pods with dilution-plate method on DG18 medium at 28 °C for 7 days.

Twenty-nine samples of leaves and pods of *Vicia faba* were gathered from various markets and farms in Qena, Egypt. Eight fungal species were isolated from the samples on DG18 medium at 28 °C. Fungal species were recovered from the samples by dilution and baiting-plate methods. The fungal-colony forming unit (CFU) in dilution-plate method was 21.05 colonies/g fresh samples and the fungal total count in baiting-plate method was 178 colonies/232 segments. Five genera comprised a total of eight species were collected from the twenty-nine samples of *Vicia faba*. *Cladosporium cladosporioides* species complex was the most common species was isolated from the samples by dilution-plate method with CFU 8.72 colonies/g fresh samples and the highest CFU was estimated in the sample no. 28 with count 5.50 colonies/g fresh samples. *Alternaria alternata* was the dominant species taking into its counts in baiting-plate method with count 118 colonies/232 segments and the highest counts were estimated in the sample nos. 13, 14, 19, 20, 21, 22, 24, 25, 26 and 28. *C. cladosporioides* species complex ranked the second position in its count in baiting plate method with count 35 colonies /232 segments and the maximum value was observed in the sample no. 26 (Figs. 1 and 2).

### Morphological characterization of *Cladosporium* sp.

Dependent upon micro- and macroscopical characteristics of the colonies, conidia, ramoconidia and presence or absence of chlamydospores, all the collected *Cladosporium* isolates were grouped into three groups; *C. cladosporioides* species complex, *C. herbarum* species complex and *C. sphaerospermum* species complex.

**Figure 3:**
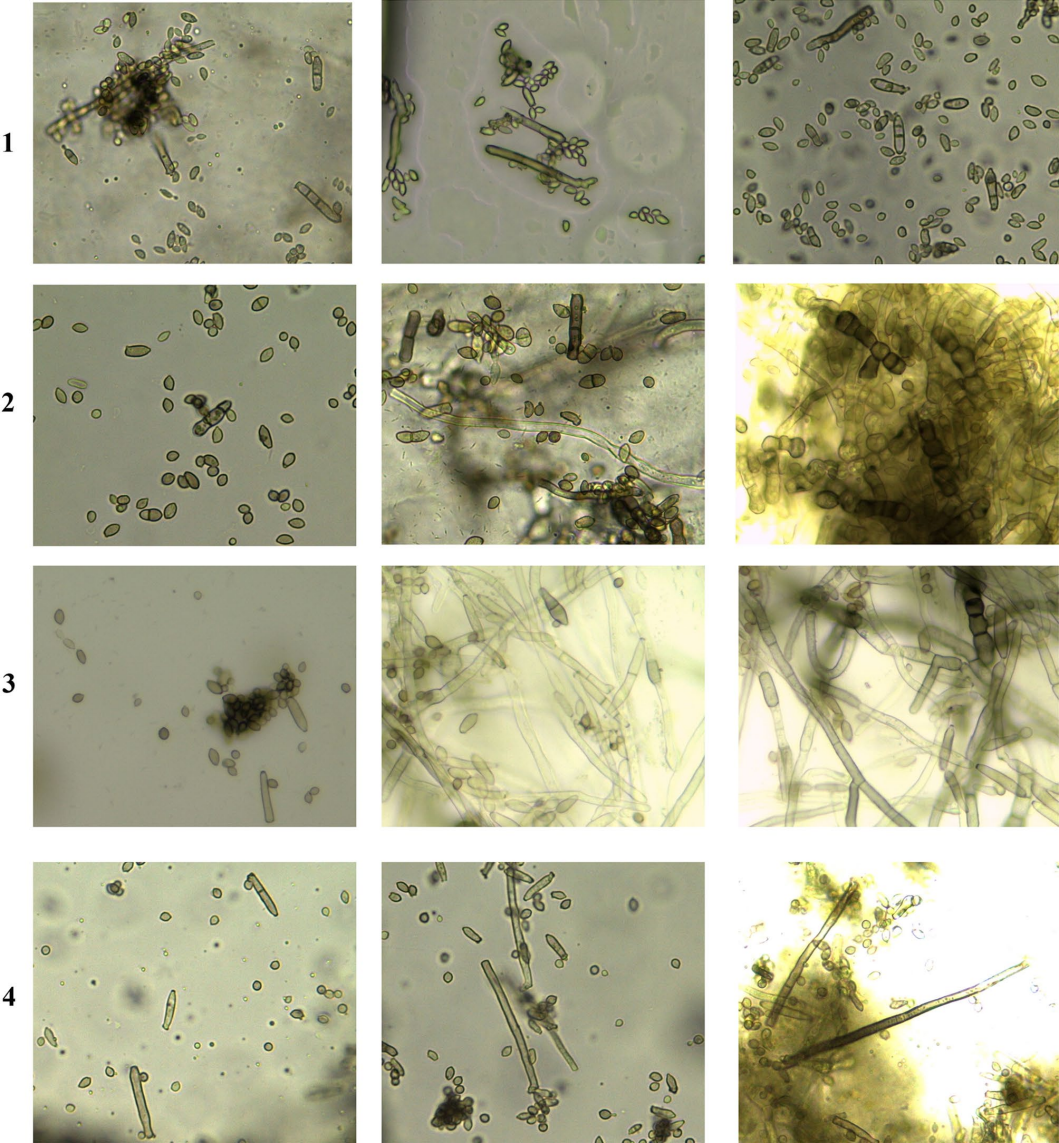
Ramoconidia, conidia, conidiophores and chlamydospores of (1): *C. cladosporioides*, (2): *C. limoniforme*, (3): *C. ramotenellum* and (4): *C. sphaerospermum.*

**Figure 4:**
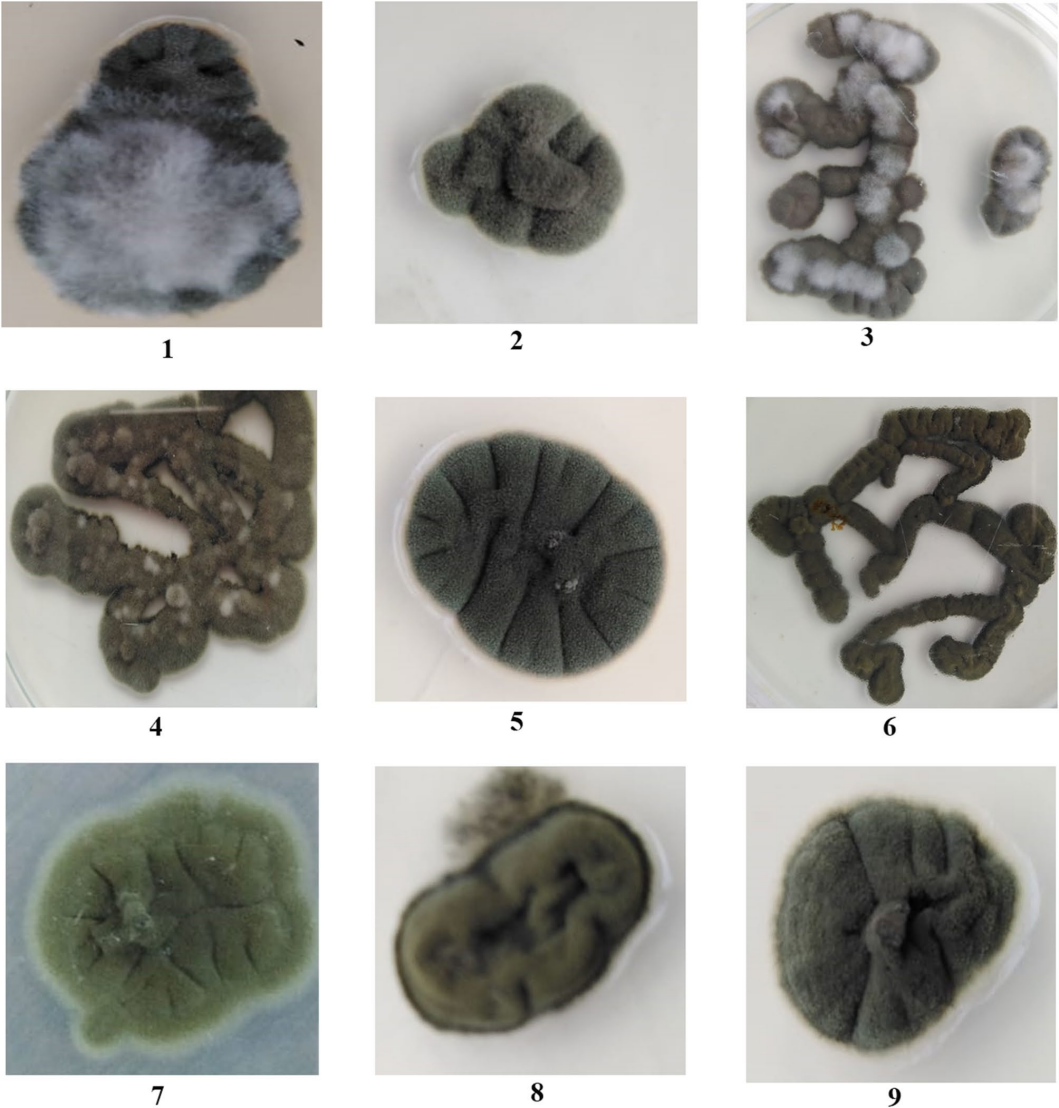
Colonies features of *Cladosporium* strains; (1–4): *C. cladosporioides*, (5–7): *C. limoniforme*, (8): *C. ramotenellum*, and (9): *C. sphaerospermum*.

Twenty-six isolates were randomly chosen to evaluate the taxonomic description by observing the micro-and macroscopical features as shown in Table 1, and Figs. 3 and 4.

**Table 1:** Morphological and cultural characteristics of *Cladosporium* sp. on PDA medium at 28 °C for 7 days.

Characteristics	*C. cladosporioides*	*C. limoniforme*	*C. ramotenellum*	*C. sphaerospermum*
**Colony**				
Color	Grey-olivaceous	Dull green due to abundant sporulation	Olivaceous due to abundant sporulation	Olive-green
Texture	Regular, feathery, white aerial mycelium sparse, diffuse, or sometimes abundantly formed. Margins white to grey-olivaceous	Glabrous. Margins regular, and white	Margin entire edge to slightly undulate, white, glabrous	Glabrous. Margins regular, and white
Reverse	Olivaceous-black and velvety	Olivaceous black, velvety	Olivaceous black, velvety	Olivaceous black, velvety
**Conidia**				
Shape	Subglobose, obovoid, ovoid to limoniform	Catenate, very numerous, ornamentation variable, obovoid to subglobose, apex rounded, attenuated towards the base, ellipsoid, limoniform, sometimes fusiform	Globose, subglobose or ovoid, obovoid or limoniform, ellipsoid, limoniform to subcylindrical	Subspherical to spherical, less often short-ovoid
Size (µm)	2.797–13.047 (length) × 0.959–3.955 (width)	2.797–13.367 × 2.143–5.928	4.34–10.075 × 2.581–3.456	4.126–5.468 × 1.517–4.098
Septum	0–1	0–1	0–1	0
**Ramoconidia**				
Shape	Ellipsoid, subcylindrical to cylindrical-oblong	Ellipsoid, fusiform to subcylindrical, slightly thickened	Ellipsoid, subcylindrical to cylindrical-oblong, sometimes swollen	Ellipsoid to cylindrical
Size (µm)	11.4–27.106 (length) × 2.035–4.422 (width)	11.391–27.139 × 2.581–6.453	15.47–30.313 × 2.396–4.418	16.405–33.153 × 2.797–4.07
Septum	0–1–2–3	0–1–2–3	0–1	0–1
**Conidiophore**				
Width (µm)	1.517–5.468	1.918–4.418	3.489–6.1	1.517–6.308
Chlamydospores	Absent	Present with abudantly	Present with rarley	Absent

#### *C. cladosporioides* species complex

Colony and texture on PDA medium were grey-olivaceous, regular, feathery and white aerial mycelium sparse, diffuse, or sometimes abundantly formed. Margin of the colony was white to grey-olivaceous. Colony reverse was olivaceous-black and velvety. Conidial shapes were subglobose, obovoid, ovoid to limoniform, with size 2.797–13.047 µm (length) × 0.959–3.955 (width) and without septa and sometimes one septum was found. Ramoconidia were observed with different shapes; ellipsoid, subcylindrical to cylindrical-oblong, with size 11.4–27.106 µm (length) × 2.035–4.422 (width), had 0–3 septate. Conidiophore was 1.517–5.468 µm in width and chlamydospores weren’t observed in this species.

#### *C. herbarum* species complex (*C. limoniforme*)

*C. limoniforme* could be identified exactly after confirmation of molecular characterization before that it inserted under the species of *C. herbarum*. Colony and texture on PDA medium were dull green due to expand and abundant sporulation. Glabrous texture was noticed, margins regular, and white. Colony reverse was olivaceous black, velvety. Shapes of conidia were catenate, very numerous, ornamentation variable, obovoid to subglobose, apex rounded, attenuated towards the base, ellipsoid, limoniform, sometimes fusiform. Size of conidia ranged 2.797–13.367 µm (length) × 2.143–5.928 µm (width), rarely one septum was found. Ramoconidial shapes were ellipsoid, fusiform to subcylindrical, slightly thickened with size 11.391–27.139 µm (length) × 2.581–6.453 µm (width), 0–3 septate was observed. Conidiophore was 1.918–4.418 µm (width) and chlamydospores were abundantly noticed in all tested isolates.

#### *C. herbarum* species complex (*C. ramotenellum*)

*C. ramotenellum* was identified morphologically under the *C. herbarum* species complex and molecular identification confirmed its name. Colony color was olivaceous due to abundant sporulation. Texture and margin entire edge to slightly undulate, white and glabrous. Reverse color olivaceous black and velvety. Shapes of conidia varied from globose, subglobose or ovoid, obovoid or limoniform, ellipsoid, limoniform to subcylindrical, with size 4.34–10.075 µm (length) × 2.581–3.456 µm (width) and sometimes one septum was observed. Shapes of ramoconidia were ellipsoid, subcylindrical to cylindrical-oblong, sometimes swollen. Ranged in the size from 15.47 to 30.313 µm (length) × 2.396–4.418 µm (width) and 0–1 septum was found. Conidiophore width was 3.489–6.1 µm and chlamydospores were present with rarley.

#### *C. sphaerospermum* species complex

Colony and texture were olive-green, glabrous. Margins regular, and white. Reverse color was olivaceous black, velvety. Conidial shape subspherical to spherical, less often short-ovoid. The size of conidia was 4.126–5.468 µm (length) × 1.517–4.098 µm (width) without septum. Ramoconidia were ellipsoid to cylindrical with size 16.405–33.153 µm (length) × 2.797–4.07 µm (width) and 0–1 septum was found. Conidiophore width was 1.517–6.308 µm (width). Chlamydospores were absent in this species.

From this study, it was observed that the main differences between the three groups of *C. cladosporioides* species complex, *C. herbarum* species complex and *C. sphaerospermum* species complex were: the colony of *C. cladosporioides* species complex was regular, feathery, white aerial mycelium sparse and diffuse, or sometimes abundantly formed. Conidia of *C. sphaerospermum* species complex was small as compared with the other two groups (up to 5.468 µm in length) and wasn’t shown septum. *C. herbarum* species complex was characterized by the formation of chlamydospores in both two species *C. limoniforme* and *C. ramotenellum* (which accurately identified by molecular technique).

### Sequencing analysis of *Cladosporium* sp.

The sequences of the tested isolates with actin gene were shown 100–97% similarity to GenBank *Cladosporium* depositing strains. The phylogenetic analysis dependent on the amplifying actin gene, showed differentiation between the studied isolates, and the three *Cladosporium* species complexes, which identified morphologically, were discriminated into four species after molecular identification.

**Figure 5:**
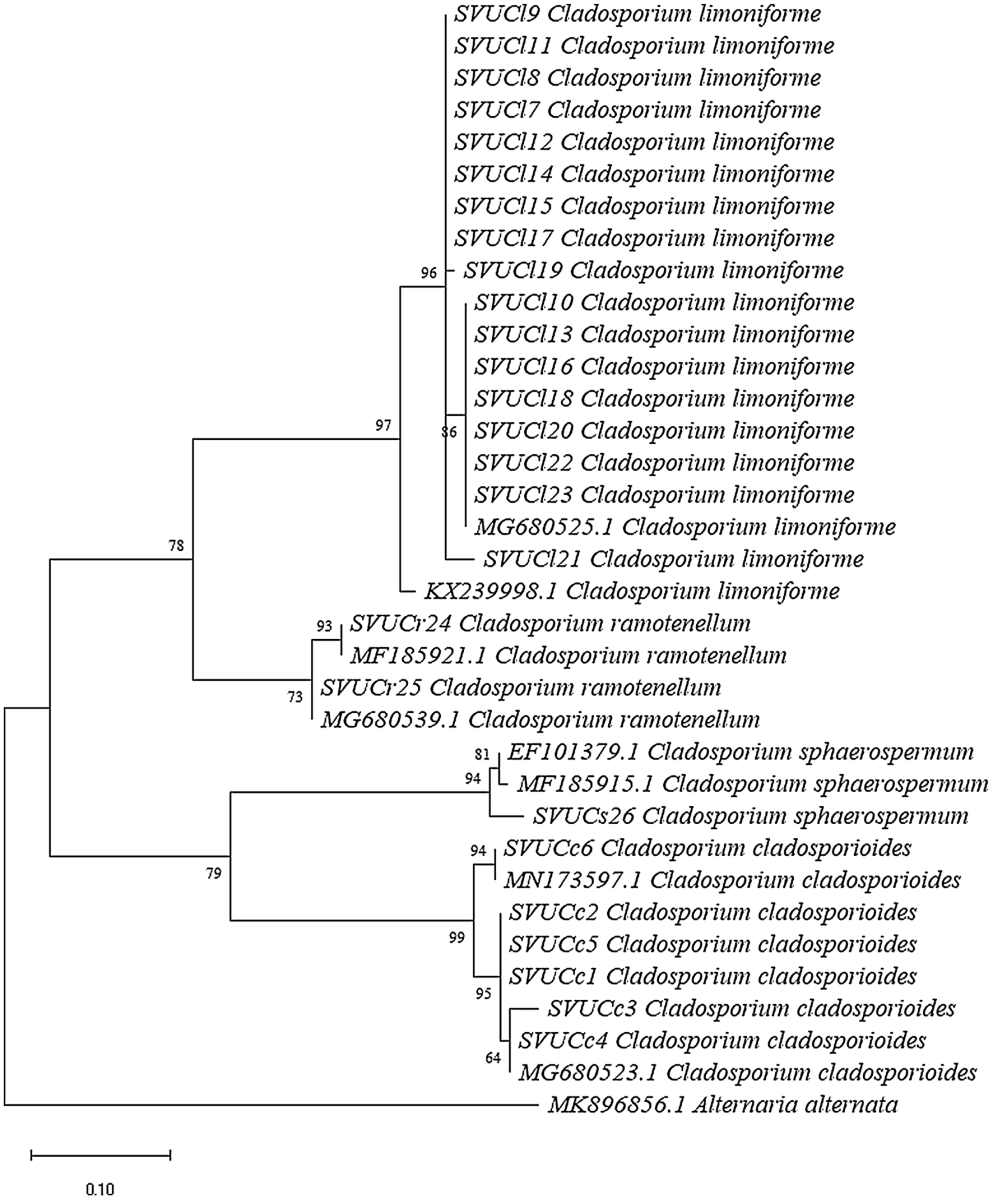
Maximum likelihood phylogenetic tree based on the analysis of ACT gene of the 26 *Cladosporium* isolates obtained from *Vicia faba* leaves and pods samples, with strains reference sequences achieved from the Genbank.

The phylogenic tree revealed that the strains could be categorized into four various clades, each representing one species. From the tested twenty-six isolates, nineteen strains which were grouped into *C. herbarum* complex in morphological characterization, here were discriminated into two species; 17 strains named as *C. limoniforme* and 2 strains *C. ramotenellum*; each species arranged in a separate clade. The first clade comprised seventeen strains of *C. limoniforme* (SVUCl7, SVUCl8, SVUCl9, SVUCl10, SVUCl11, SVUCl12, SVUCl13, SVUCl14, SVUCl15, SVUCl16, SVUCl17, SVUCl18, SVUCl19, SVUCl20, SVUCl21, SVUCl22 and SVUCl23) clustered together with KX239998.1 and MG680525.1 obtained from the Genbank with bootstrap 97. The second clade consisted from two strains of *C. ramotenellum* (SVUCr24 and SVUCr25), which clustered together with MF185921.1 and MG680539.1, which were achieved from the Genbank with bootstrap 73. The third clade formed from the single strain of *C. sphaerospermum* which clustered with two strains obtained from the Genbank (EF101379.1 and MF185915.1) named also as *C. sphaerospermum* with bootstrap 94. Six strains were belonged to the *C*. *cladosporioides* complex in morphological identification, discriminated molecularly as *C*. *cladosporioides* (SVUCc1, SVUCc2, SVUCc3, SVUCc4, SVUCc5 and SVUCc6) in the fourth clade and grouped with two strains obtained from Genbank; named as *C*. *cladosporioides* with accession numbers MG680523.1 and MN173597.1, and bootstrap value 99 (Fig. 5).

### Pathogenic activity of *Cladosporium* sp.

The pathogenic effect of 26 strains of *Cladosporium* belonged to four species were tested. All the studied strains were able to cause leaf spot infections on *Vicia faba* leaves with different levels.

**Figure 6:**
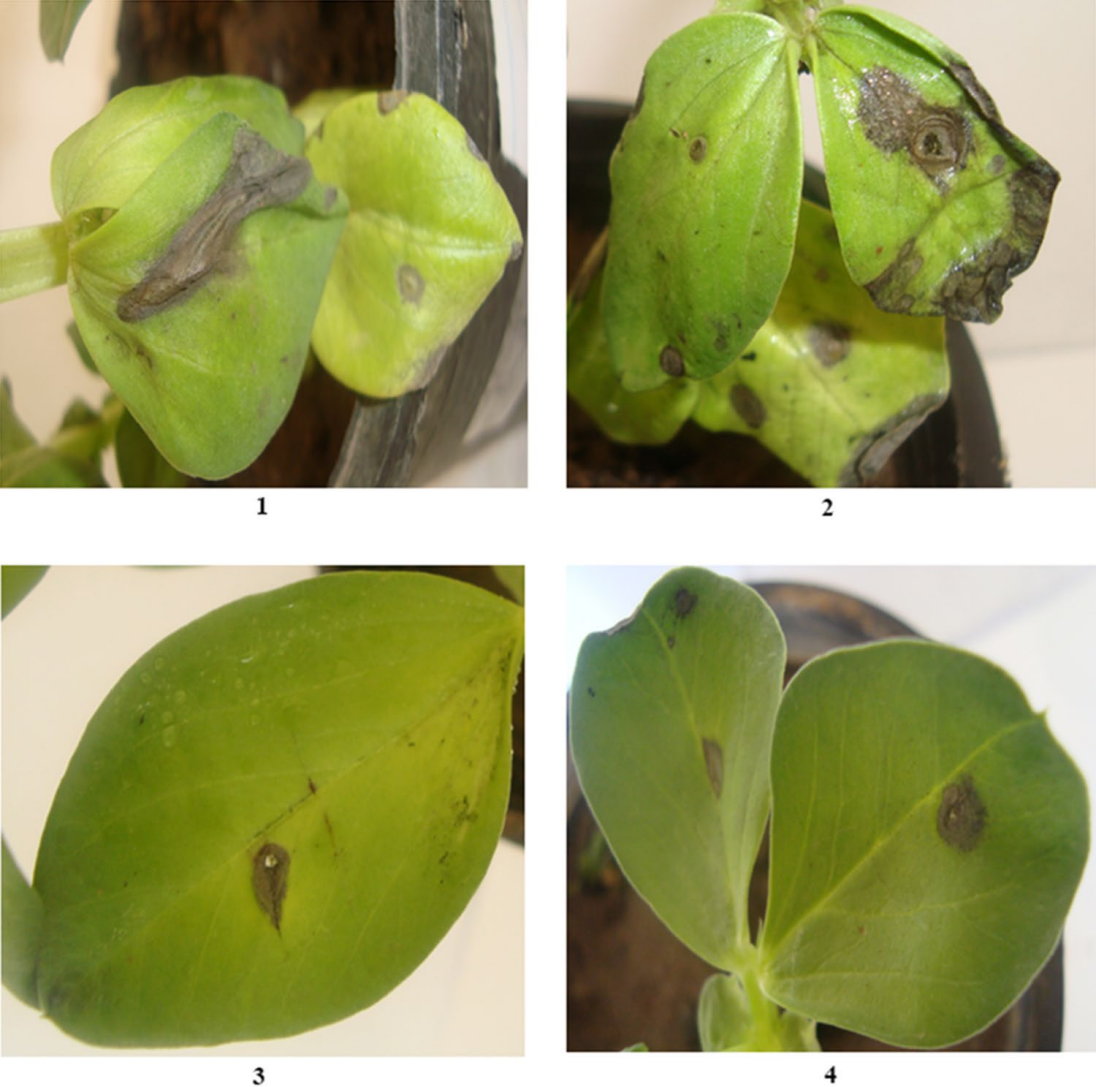
Lesions caused by *Cladosporium* species on leaves of *Vicia faba*; (1): *C. cladosporioides*, (2): *limoniforme*, (3): *C. ramotenellum*, and (4): *C. sphaerospermum.*

The two strains of *C. ramotenellum* (SVUCr24 and SVUCr25) were significant moderately virulent, which caused lesions,and infected 50–75% of the leaves surface. Eleven strains of *C. limoniforme* (SVUCl7, SVUCl9, SVUCl10, SVUCl11, SVUCl12, SVUCl15, SVUCl16, SVUCl18, SVUCl19, SVUCl21 and SVUCl23) were significant moderately virulent (lesion on 50–75% of infected leaves). Two strains of *C. limoniforme* (SVUCl17 and SVUCl22) were significant highly virulent as the lesions appeared more than 75% of the leaf surface. The other four species of *C. limoniforme* (SVUCl8, SVUCl13, SVUCl14 and SVUCl20) were non-significant hypovirulent, lesions were recorded on less than 50% of infected leaves. SVUCs26 of *C. sphaerospermum* also was non-significant hypovirulent. Five strains of *C*. *cladosporioides* (SVUCc1, SVUCc2, SVUCc3, SVUCc4 and SVUCc5) were significant moderately virulent. The other one strain of *C*. *cladosporioides* (SVUCc6) was non-significant hypovirulent (Table 2 and Fig. 6).

**Table 2:** Codes, accession numbers, and pathogenic effects of *Cladosporium *strains on *Vicia faba* plants.

Code of isolate	Isolates name	Mean of pathogenic activity	Accession number
SVUCc1	*Cladosporium cladosporioides*	B*	MZ164887
SVUCc2	*C*. *cladosporioides*	B*	MZ164888
SVUCc3	*C*. *cladosporioides*	B*	MZ164889
SVUCc4	*C*. *cladosporioides*	B*	MZ164890
SVUCc5	*C*. *cladosporioides*	B*	MZ164891
SVUCc6	*C*. *cladosporioides*	A	MZ164892
SVUCl7	*C. limoniforme*	B*	MZ164893
SVUCl8	*C. limoniforme*	A	MZ164894
SVUCl9	*C. limoniforme*	B*	MZ164895
SVUCl10	*C. limoniforme*	B*	MZ164896
SVUCl11	*C. limoniforme*	B*	MZ164897
SVUCl12	*C. limoniforme*	B*	MZ164898
SVUCl13	*C. limoniforme*	A	MZ164899
SVUCl14	*C. limoniforme*	A	MZ164900
SVUCl15	*C. limoniforme*	B*	MZ164901
SVUCl16	*C. limoniforme*	B*	MZ164902
SVUCl17	*C. limoniforme*	C*	MZ164903
SVUCl18	*C. limoniforme*	B*	MZ164904
SVUCl19	*C. limoniforme*	B*	MZ164905
SVUCl20	*C. limoniforme*	A	MZ164906
SVUCl21	*C. limoniforme*	B*	MZ164907
SVUCl22	*C. limoniforme*	C*	MZ164908
SVUCl23	*C. limoniforme*	B*	MZ164909
SVUCr24	*C. ramotenellum*	B*	MZ164910
SVUCr25	*C. ramotenellum*	B*	MZ164911
SVUCs26	*C. sphaerospermum*	A	MZ164912

Pathogenicity rating, A: hypovirulent; B: moderately virulent and C: virulent. *Means were significantly at p < 0.05.

### Inhibition growth of *Cladosporium* isolates by *C. globosum*

**Figure 7:**
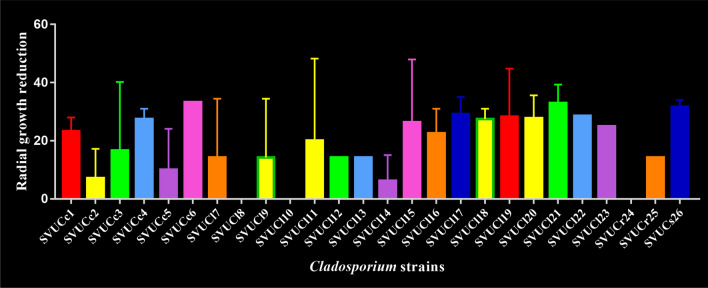
Inhibitory effect of *C. globosum* on the growth of *Cladosporium* strains.

*C. globosum* clearly inhibited the growth of *Cladosporium* isolates in dual test plates at ranges from 12.5 to 40 mm. The maximum inhibition growth was observed in isolate of SVUCl11, and the lowest value was noticed in SVUCl14. The growth of SVUCl8, SVUCl10 and SVUCr24 weren’t inhibited by *C. globosum*. All isolates of *C. cladosporioides* were inhibited by *C. globosum* at levels from 14.28 to 33.33 mm. SVUCr25 was inhibited by *C. globosum* at 14.28 mm. Isolate of SVUCs26 was inhibited at 31.67 mm (Fig. 7).

## Discussion

In this study eight species were isolated from leaves and pods of *Vicia faba*, they were identified by morphological and molecular methods, and the most common species in the count were *Alternaria alternata* and *Cladosporium cladosporioides*. *C. oxysporum* and *C. sphaerospermum* were recorded by Paul and Yu^29^; the two species of *Cladosporium* were isolated from the needles of pine trees (*Pinus* sp.) in Korea. According to Elwakil et al.^30^, faba bean seeds were inhabited by 13 different fungal genera, among which were *Aspergillus*, *Penicillium*, *Alternaria*, *Botrytis*, *Cladosporium*, *Epicoccum*, *Fusarium*, and *Rhizopus*. Our result has been in agreement with Saleem et al.^31^ who estimated that fourteen species were isolated from diseased leaves of broad bean, collected fungal species: *Alternaria alternata*, *Cladosporium cladosporioides* and *C. sphaerospermum*. Pszczółkowska et al.^32^ reported that *Alternaria alternata*, *Aspergillus* sp. *Cladosporium cladosporioides* were determined among sixteen fungal species isolated from faba bean seeds harvested in the regions of Warmia and Mazury and Lower Silesia in Poland.

In this study *C. cladosporioides* showed morphological characteristics were in agreement and in the ranges described with Bensch et al.^9^ who reported that ramoconidia cylindrical-oblong, 15–50 × 2.5–5 μm, with up to three septa, pale olivaceous brown. Secondary ramoconidia ellipsoid, subcylindrical to cylindrical-oblong, 7–38 × 2–6 μm (0–1) septate, occasionally with two septa. Conidia numerous, catenate, small terminal conidia obovoid, ovoid to limoniform, subglobose, 3–7 × 1.5–3 μm aseptate, intercalary conidia limoniform, ellipsoid-ovoid, sometimes fusiform or subcylindrical, 5–14.5 × 2–4 μm, aseptate. In this study, it was found that the colony was more grey feathery texture formed in all *C. cladosporioides* isolates after five days of incubation.

*C. limoniforme* morphological features were shown partially similarities to those described with Bensch et al.^11^ who reported that colonies on PDA were smoke-grey, dark grey olivaceous to iron-grey, reverse olivaceous black to iron-grey, velvety to granular; margins broad, regular, white, glabrous to feathery; aerial mycelium diffuse, sometimes found in colony center; growth regular, flat. In this report, this species was characterized by the abundantly formation of chlamydospores and also the properties of ramoconidia and secondary ramoconidia showed differences in the size from those described by Bensch et al.^11^ who stated that ramoconidia 15–50 μm long, (0–1) septate, base 2–3 μm wide. Secondary ramoconidia subcylindrical to fusiform, ellipsoid, 8–30 × 2.5–4 μm, 0–1 septate, pale olivaceous brown or pale brown, surface ornamentation variable, loosely verruculose, sometimes spiny or irregularly rough-walled, walls unthickened. 

In this study, some of the morphological properties of *C. ramotenellum* were different from other studies: ramoconidia up to 30.313 μm in length and 4.418 μm in width; aseptate ramoconidia were abundantly and one septum sometimes was formed. Bensch et al.^9^ reported that ramoconidia were cylindrical-oblong, 0–1 septate, rarely up to 4 septate, fluctuated between 47 μm long and 2–4 μm wide. Polymorphous conidia were recorded in this study: 4.34–10.075 μm in length × 2.581–3.456 μm in width; 0–1 septate. Bensch et al.^9^ recorded that conidia were catenate, in branched chains, straight, sometimes slightly curved, small terminal conidia numerous, globose, subglobose or obovoid or ovoid, or limoniform, 2.5–7 × 2–4.5 μm, aseptate, without distal hilum or with a single apical scar; intercalary conidia subcylindrical to ellipsoid, 8–15 × 3–4.5 μm, 0–1 septate. In this study, chlamydospores were present with rarley.

Morphological characterization of *C. sphaerospermum* in this study partially agreed with the description obtained with Paul and Yu^29^ who found that conidia were spherical, ellipsoidal to cylindrical with sounded ends, with dimensions of 2.0–8.0 × 2.0–4.0 μm and secondary ramoconidia had 0–4 septum with size were 8.5–20.0 × 3.0–6.0 μm, but in this study, conidial size were 2.797–13.047 (length) × 0.959–3.955 (width), sometimes with one septum; ramoconidial size were 11.4–27.106 (length) × 2.035–4.422 (width). 

Identification of *Cladosporium* with morphological characterization is partially difficult due to the high morphological similarities between species^33^. This finding was confirmed while morphological group classification was carried out. This has observed because the morphological features grouped the collected isolates into three groups; after molecular confirmation they grouped into four species, because molecular identification is more accurate and could differentiate the fungi under species level. The phylogenetic analysis was done by amplifying of actin gene. As reported with many researchers, actin gene could discriminate between species of *Cladosporium*^12,13,33,34^. In this study, three groups were observed morphologically: *C. cladosporioides* complex species, *C. herbarum* complex, *C. sphaerospermum* complex; they were discriminated into four species: *C. cladosporioides, C. limoniforme, C. ramotenellum* and *C. sphaerospermum.*

One hundred percent of *Cladosporium* strains were pathogenic by appearing of lesions to inoculated leaves of *Vicia faba*; 20 strains were significant and the other six strains weren’t as compared with non-infected leaves. It was reported by Snyder^35^ that primary infection by *Cladosporium* develops through the soil or from the seeds, or from diseased pods that showed discoloured spots, and these when sown appeared a various percentage of infected seedlings. Surface sterilization of seeds and pods was not preventing disease. The existence of the fungus in the pods perhaps cause hair-like propagation of the inner tissue, resulting in the formation of felty and white patches extending into the pod cavity. Abdel-Motaal et al.^36^ reported that *Cladosporium herbarum* caused disease on *Hyoscyamus muticus* plant, firstly developed after 2 weeks as white spots, which increased and became after 3 weeks to brown. Our result has been in disagreement with Saleem et al.^31^ who found that *Cladosporium cladosporioides* and *C. sphaerospermum* showed negative pathogenic effects on the leaves of broad bean plants and the inoculated leaves couldn’t develop any lesions. It has been proven by Temperini et al.^34^ the ability of *C. herbarum* complex species and *C. cladosporioides* complex species to cause lesions in healthy pears.

The growth of all isolates of tested *Cladosporium* were inhibited by *C. globosum* except three isolates didn’t show inhibition. As reported with Wang et al.^37^ *C. globosum* had various ranging inhibitory effects on plant pathogenic bacteria and fungi. When *C. globosum* intersected with pathogens on the same cultured plates, plant pathogens were surrounded by it, and the margins of colonies collapsed. *Chaetomium globosum* colonized the nutritious space and penetrating pathogens mycelia, the pathogens stopped growing, spores were reduced in the number, abnormal branches increased, mycelial walls damaged and all cell contents released and finally die.

## Conclusion

In this study, we could prove that the genus of *Cladosporium* has complex species, in addition to morphological characterization, molecular sequences are required to discriminate between closely related species. The pathogenic effect of *Cladosporium* strains was evaluated and the using of biocontrol method to prevent its pathogenicity was determined successfully by *C. globosum.*

## Supplementary Information

Supplementary Information 1.

Supplementary Information 2.

Supplementary Information 3.

Supplementary Information 4.

Supplementary Information 5.

## Data Availability

Sequence data have been submitted to GenBank, https://www.ncbi.nlm.nih.gov/nucleotide/, and we attached the text file sequences, the submission text bankit, also we waite the accession numbers.
